# The role of the calibrated automated thrombogram in neonates: describing mechanisms of neonatal haemostasis and evaluating haemostatic drugs

**DOI:** 10.1007/s00431-021-04196-8

**Published:** 2021-07-20

**Authors:** Claire A. Murphy, Elaine Neary, Daniel P. O’Reilly, Sarah Cullivan, Afif EL-Khuffash, Fionnuala NíAinle, Patricia B. Maguire, Naomi McCallion, Barry Kevane

**Affiliations:** 1 Department of Paediatrics, Royal College of Surgeons in Ireland, Dublin, Ireland; 2 Department of Neonatology, Rotunda Hospital, Dublin, Ireland; 3 Department of Neonatology, Liverpool Women’s Hospital, Liverpool, UK; 4 Department of Health and Life Sciences, University of Liverpool, Liverpool, UK; 5 Department of Haematology, Mater Misericordiae University Hospital, Dublin, Ireland; 6 Department of Haematology, Rotunda Hospital, Dublin, Ireland; 7 School of Medicine, University College Dublin, Dublin, Ireland; 8 Conway-SPHERE Research Group, Conway Institute, University College Dublin, Dublin, Ireland

**Keywords:** Haemostasis, Neonates, Platelets, Thrombin, Bleeding, Thrombosis

## Abstract

Premature infants are at high risk of haemorrhage and thrombosis. Our understanding of the differences between the neonatal and adult haemostatic system is evolving. There are several limitations to the standard coagulation tests used in clinical practice, and there is currently a lack of evidence to support many of the transfusion practices in neonatal medicine. The evaluation of haemostasis is particularly challenging in neonates due to their limited blood volume. The calibrated automated thrombogram (CAT) is a global coagulation assay, first described in 2002, which evaluates both pro- and anti-coagulant pathways in platelet-rich or platelet-poor plasma. In this review, the current applications and limitations of CAT in the neonatal population are discussed.

*Conclusion*: CAT has successfully elucidated several differences between haemostatic mechanisms in premature and term neonates compared with adults. Moreover, it has been used to evaluate the effect of a number of haemostatic drugs in a pre-clinical model. However, the lack of evidence of CAT as an accurate predictor of neonatal bleeding, blood volume required and the absence of an evidence-based treatment algorithm for abnormal CAT results limit its current application as a bedside clinical tool for the evaluation of sick neonates.

**Table:** 

**What is Known:** • *The Calibrated automated thrombogram (CAT) is a global coagulation assay which evaluates pro- and anti-coagulant pathways.* • *CAT provides greater information than standard clotting tests and has been used in adults to evaluate bleeding risk.*	
**What is New:** • *This review summarises the physiological differences in haemostasis between neonates and adults described using CAT.* • *The haemostatic effect of several drugs has been evaluated in neonatal plasma using CAT.*

**What is Known:**

• *The Calibrated automated thrombogram (CAT) is a global coagulation assay which evaluates pro- and anti-coagulant pathways.*

• *CAT provides greater information than standard clotting tests and has been used in adults to evaluate bleeding risk.*

**What is New:**

• *This review summarises the physiological differences in haemostasis between neonates and adults described using CAT.*

• *The haemostatic effect of several drugs has been evaluated in neonatal plasma using CAT.*

## Background

Preterm infants experience haemorrhage, particularly intraventricular haemorrhage (IVH), while full-term neonates typically do not [1]. Neonates have reduced levels of coagulation factors [2, 3] and hypo-reactive platelets in vitro [4–6], but higher levels of von Willebrand factor (VWF) with larger polymers and a higher haematocrit help maintain haemostasis [2, 3, 7].

Prothrombin time (PT) and activated partial thromboplastin time (APTT) are the standard tests used to evaluate haemostasis. Both are prolonged in preterm infants [8, 9], although there is no correlation between PT/APTT and risk of developing IVH [8, 10, 11]. PT and APTT only evaluate time to initial clot formation, not overall clot formation [12, 13]. Moreover, standard clotting tests do not evaluate for hypercoagulability [14]. Despite these limitations, they are often used to guide transfusion of blood products to correct perceived coagulation disturbances in non-bleeding neonates.

**Fig. 1:**
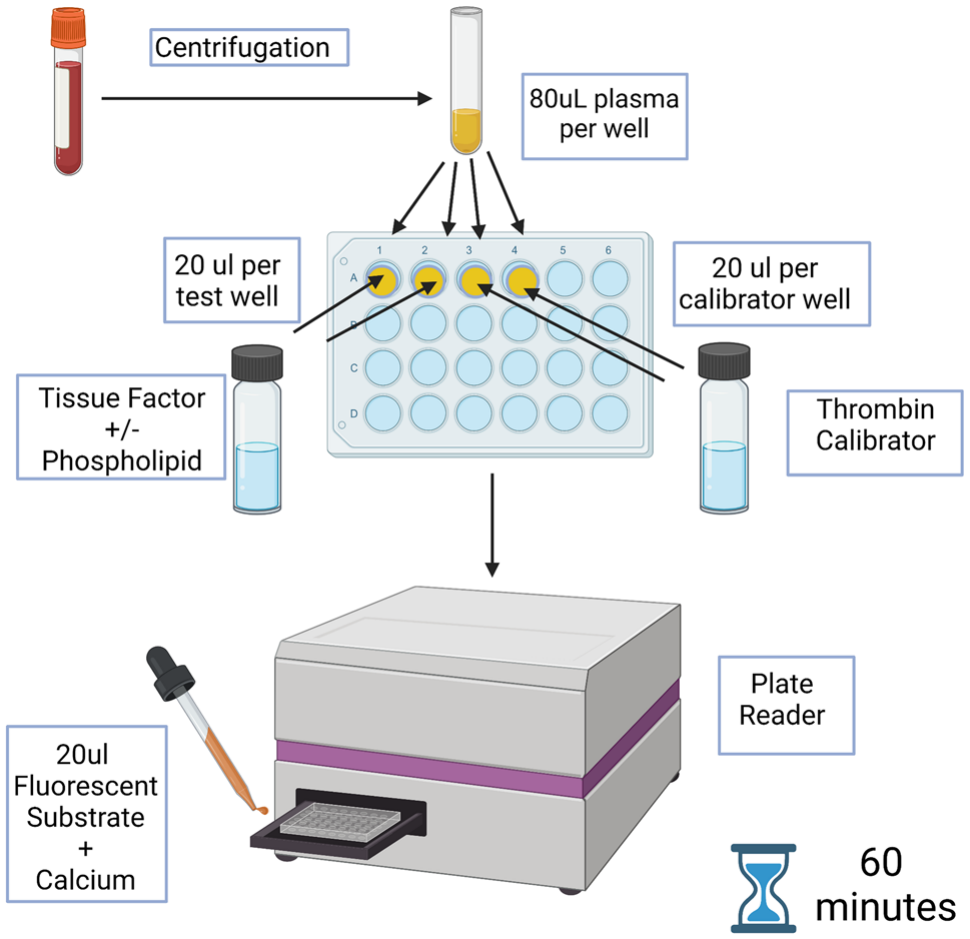
The standard process for performing CAT in plasma (in duplicate) (Image created with BioRender.com)

The calibrated automated thrombogram (CAT) is a global coagulation assay, which measures thrombin generation by incubating plasma with a thrombin-specific fluorogenic substrate following activation of coagulation (Fig. 1) [13]. CAT assesses overall haemostatic balance, evaluating activity of endogenous procoagulant and anticoagulant pathways, particularly important in neonates, where both are reduced [3]. CAT is performed in platelet-poor (PPP) or platelet-rich plasma (PRP). In PRP, CAT can evaluate the impact of platelet number and platelet function on thrombin generation [15].

**Fig. 2:**
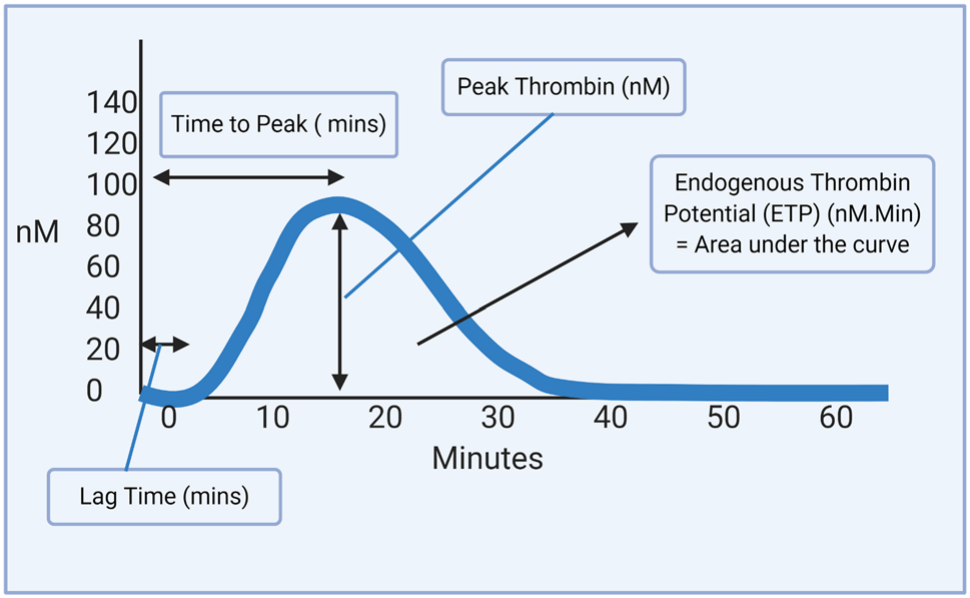
A standard thrombin generation curve. The lag time, time to peak thrombin, endogenous thrombin potential and peak thrombin are displayed. (Image created with BioRender.com)

**Fig. 3:**
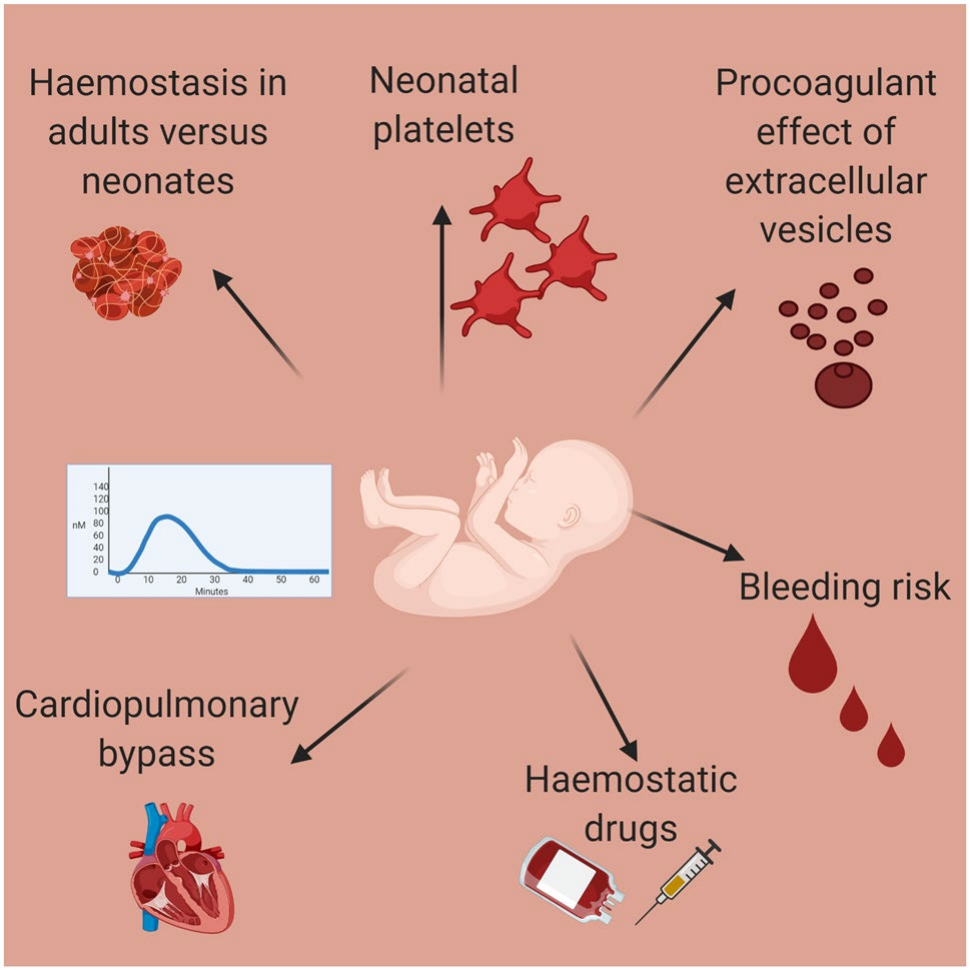
The neonatal applications of CAT. CAT has been used to evaluate the differences in secondary haemostasis in preterm and term infants compared with adults and the relative haemostatic effect of platelets and extracellular vesicles in neonates. The use of CAT as a mechanism to test haemostatic therapies in a pre-clinical model and in infants at high risk of haemorrhage, particularly those undergoing cardiopulmonary bypass are also described. (Image created with BioRender.com)

The CAT parameters are described in Fig. 2. The lag time is the time from the beginning of the experiment until 10 nM of thrombin is produced [15]. Time to peak thrombin represents the propagation phase. Peak thrombin is the maximum amount of thrombin produced, while the endogenous thrombin potential (ETP) represents the total amount of thrombin produced during the clotting process. ETP is the parameter most predictive of bleeding or thrombosis [16]. A shortened lag time and increased ETP/peak thrombin suggests a hypercoagulable status, while a prolonged lag time and reduction in ETP/peak thrombin suggests a hypocoagulable state. In this review, the applications and limitations of CAT in neonates are described (Table 1; Fig. 3).

**Table 1:** Summary of the included studies which have used CAT in neonates

Author	Year	Plasma	Neonatal blood source	Neonatal population	Control	Number of participants	Main neonatal CAT findings
Fritsch et al. [24]	2006	PPP	UCB	Term infants	Adults	Term infant = 28 Neonate with FVIII deficiency = 1 Adult = not described	Neonates had a shortened lag time and TTP, but reduced ETP and peak thrombin compared with adult PPP FVIII deficiency (in vitro) caused a slight prolongation in lag time and TTP but no reduction in peak thrombin. Increasing TFPI levels caused a prolongation in lag time and time to peak while increasing AT caused a reduction in peak height and ETP
Cvirn et al. [20]	2007	PPP	UCB	Term infants	Adults	Term infant = 16 Adult = 17	Neonates had a shortened lag time and TTP, but reduced ETP and peak thrombin compared with adult PPP Melagatran exhibited distinctly different patterns of sensitivity in adult and neonatal PPP
Schweintzger et al. [21]	2011	PPP	UCB	Term infants	Adults	Term infant = 31 Adult = 28	Neonates had a shortened lag time and TTP but reduced ETP and peak thrombin compared with adult PPP TF-EVs had a greater haemostatic effect in neonates
Franklin et al. [19]	2016	PPP	Peripheral (Arterial line)	Term infants undergoing CPB	Adults	Term infant = 15 Adult = 20	Neonates had a shortened lag time but reduced peak thrombin compared with adult PPP In neonates after reversal of CPB, lag time remains prolonged, but peak thrombin is increased Lower doses of both 4f-PCCs tested were sufficient to increase peak thrombin in neonates. Only 4f-PCC containing FVIIa reduced lag time to pre-CPB levels
Schlagenhauf et al. [22]	2017	PPP	UCB	Term infants	Adults	Term infant = 17 Adult = 12	Neonates had a shortened lag time and TTP, but this study found no difference in ETP or peak thrombin compared with adult PPP Polyphosphate had a reduced relative impact on thrombin generation parameters in neonates, but lower concentrations of polyphosphate were required to exert maximal effect
Haidl et al. [23]	2019	PPP	UCB	Term infants	Adults	Term infant = 30 Adult = 20	Neonates had a shortened lag time and TTP, but reduced ETP and peak thrombin compared with adult PPP
Haidl et al. [25]	2019	PRP	UCB	Term infants	Adults	Term infant = 10 Adult = 10	Neonates had a shortened lag time and TTP, a reduced ETP but higher peak thrombin compared with adult PRP TG in neonates in not dependent on platelet count NovoSeven® altered clot dynamics but not ETP, was not dependent on platelet count and the dose response was similar in neonates and adults
Tripodi et al. [29]	2008	PPP	UCB and peripheral	Preterm infants (30–37 weeks)	Term infants	Preterm UCB = 55 Preterm peripheral = 19 Term UCB = 109 Term peripheral = 37	ETP was significantly higher in preterm infants
Neary et al. [8]	2015	PPP	UCB	Preterm infants (24–30 weeks)	Term infants	Preterm infant = 15 Term infant = 13	Lag time and TTP were significantly shorter in the preterm group
Tripodi et al. [9]	2020	PPP	Peripheral	Preterm infants (< 1500 g)	Term infants	Preterm infant = 87 Term infant = 64	A procoagulant imbalance in preterm infants compared with term infants
Bernhard et al. [38]	2009	PRP*	UCB	Term infants	Adults	Term infant = 12 Adult = 12	Neonatal and adult platelets supported thrombin generation comparably
Peterson et al. [40]	2018	PRP	Peripheral (arterial line)	Term infants undergoing CPB		Term infants = 44	Post-op CAT correlated better with TFCK ratios than immediately post CPB reversal Peak thrombin inversely correlated with high TFCK ratios (blood samples with the highest heparin activity)
Guzzetta et al. [58]	2013	PPP	Peripheral	Term infants undergoing CPB		Term infant = 11	After reversal of CPB, lag time remains prolonged but peak thrombin is increased Ex vivo addition of NovoSeven® reduced the lag time only. 3f-PCC reduced lag time and significantly increased peak thrombin and velocity index
Ghirardello et al. [62]	2020	PPP	Peripheral	Preterm infants with IFALD	Preterm infants without IFALD	IFALD = 32 No IFALD = 60	Preterm infants with IFALD had similar ETP to infants without IFALD

*3f-PCC* three factor prothrombin complex concentrate, *4f-PCC* four factor prothrombin complex concentrate, *AT* antithrombin, *CAT* calibrated automated thrombography, *CPB* cardiopulmonary bypass, *ETP* endogenous thrombin potential, *EV* extracellular vesicle, *IFALD* intestinal failure associated liver disease, *PPP* platelet poor plasma, *PRP* platelet-rich plasma, *TF* tissue factor, *TFCK* thrombin-initiated Fibrin Clot Kinetics *TFPI* tissue factor pathway inhibitor, *TG* thrombin generation, *TTP* time to peak thrombin, *UCB* umbilical cord blood

## Physiological haemostasis

Physiological coagulation is triggered by exposure of sub-endothelial tissues at the site of vascular injury [17]. The extrinsic pathway is activated by sub-endothelial tissue factor (TF) and circulating activated FVII, generating thrombin via the common pathway and activating the intrinsic pathway. Thrombin cleaves fibrinogen to fibrin, thus stabilising the clot. Platelets also become activated at the site of vascular injury through interactions with sub-endothelial tissues, von Willebrand factor, fibrinogen, and agonists such as thrombin. To limit coagulation to the site of injury, there are several inhibitory mechanisms, including tissue factor pathway inhibitor (TFPI), antithrombin, protein C pathway and fibrinolysis. Pathological changes to pro- or anti-coagulant activity can result in excessive bleeding or thrombosis [18].

## Haemostasis in healthy term neonates

While FVIII and von Willebrand factor levels are usually within or above the healthy adult range at birth, levels of most other procoagulant factors are lower [2, 3]. Term infants do not display increased bleeding tendency despite these reduced levels. Levels of several anticoagulant factors (antithrombin, protein C, protein S) are also reduced [2, 3].

Six studies used CAT to characterise thrombin generation in platelet-poor plasma from term newborns compared with adults [19–24]. All demonstrated a significantly shortened lag time and time to peak in neonates. However, neonates had significantly reduced ETP and peak thrombin compared with adults, although one study showed no difference [22]. Similar findings were described in neonatal platelet-rich plasma [25].

It is hypothesised that this reduction in lag time, and time to peak in neonates (suggestive of a hypercoagulable state) is due to reduced TFPI, and that the reduction in ETP and peak thrombin (suggestive of a hypocoagulable state) is due to lower levels of pro-coagulant factors (particularly factor II) [23, 26], which itself might be further offset by a reduction in physiological anti-coagulant factors such as antithrombin [3].

## Haemostasis in the preterm infant

Preterm infants are at high risk of haemorrhage and thrombosis [27, 28] and have reduced levels of procoagulant (FIX, FXI, FXII and fibrinogen) and anti-coagulant factors (antithrombin, protein C and protein S) compared with term neonates [2].

Three studies evaluated CAT in PPP from preterm compared with term infants. In preterm infants > 30 weeks gestation, ETP was higher than term controls [29]. Neary et al. demonstrated a significantly shorter lag time and time to peak in umbilical cord blood in preterm infants (24–30 weeks gestation), but found no difference in ETP or peak thrombin between groups [8].

Most recently, Tripodi et al. characterised thrombin generation in peripheral blood in very low birth weight (VLBW) infants < 1500 g. VLBW infants had higher ETP than term controls [9]. However, infants < 30 weeks gestation had significantly lower ETP than infants > 30 weeks (unfortunately, no comparison to term ETP was provided). There was no difference in ETP between small for gestational age (SGA) and appropriately grown infants. These findings in SGA infants replicate findings by Sokou et al. using thromboelastography (TEG), an alternative global coagulation assay [30].

Using thrombomodulin (TM), a key regulator of the protein C pathway, ETP-TM ratio was higher in preterm infants, suggesting a resistance to Protein C and thus a procoagulant imbalance in preterm plasma [9]. Interestingly, the presence of a procoagulant imbalance in preterm plasma may predispose to IVH, possibly due to an increased risk of venous infarction and venous haemorrhage. This hypothesis is supported by data describing increased IVH risk associated with hereditary thrombophilia [10]. Moreover, a study using TEG demonstrated a trend towards hypercoagulability in premature infants with IVH, compared to those without [31]. While these findings are not conclusive, they highlight the inability of standard clotting tests (PT/APTTs) to accurately reflect the true complexity of haemostatic balance in vivo.

## Evaluating the effect of neonatal platelets

Although thrombocytopenia is common in sick neonates [32], platelet number is a poor predictor of severe haemorrhage [33]. In the PlaNeT 2 study, more liberal platelet transfusions were associated with a significantly higher incidence of mortality or severe haemorrhage, in thrombocytopenic infants [34]. The cause of this is not yet clear [35], although several differences exist between neonatal and adult platelets [4, 7]. While neonatal platelets are hyporesponsive to multiple agonists in vitro [4–6], the in vivo haemostatic consequences of this are poorly understood.

CAT in PRP is performed using a reagent which contains tissue factor only (without a source of exogenous phospholipids). This renders the assay dependent upon the phospholipid content of PRP.

Haidl et al. compared thrombin generation in PRP from term cord blood and adults [25]. In neonatal PRP, there were no differences in any thrombin generation parameters at platelet counts of 10,000/µL and 100,000/µL, suggesting that neonatal thrombin generation is not dependent on absolute platelet number. In contrast, thrombin generation in adult PRP is dependent on platelet count [15, 36]. CAT was evaluated in TFPI-depleted adult PPP, following the addition of high or low concentrations of TFPI, and varying concentrations of platelets [25]. Lower levels of TFPI (to represent neonatal plasma) were associated with lower platelet dependency of thrombin generation. Although endogenous TFPI activity levels in cord blood and adult samples were not described and would have been useful to confirm the hypothesis, reduced TFPI activity in neonates has been reported [26, 37].

The respective effects of neonatal and adult platelets on thrombin generation were evaluated by CAT, following the addition of platelets (neonatal/adult) to PPP (neonatal/adult) [38]. Newborn and adult platelets supported thrombin generation comparably. This suggests that CAT parameters were primarily determined by the plasma present (neonatal/adult). These results differ from a similar study using TEG, which found that the “transfusion” of neonatal platelets resulted in a shorter reaction time in both neonatal and adult blood, while the transfusion of adult platelets to cord blood resulted in a greater maximal amplitude and clot firmness, compared to neonatal platelets [39].

Schlagenhauf et al. demonstrated that upon stimulation, neonatal platelets release fewer inorganic polyphosphates, a pro-coagulant substance released from dense granules of activated platelets [22]. Using CAT, exogenous polyphosphates had a lower relative impact on thrombin generation parameters in neonatal PPP, but exerted their maximal effect at lower concentrations than in adults. Lower TFPI levels rendered neonates more sensitive to the effect of polyphosphate, while limiting the potential impact.

Different PRP preparation techniques were used in the platelet studies, which may explain some variability in the findings. Haidl et al. centrifuged whole blood at 200 × g for 10 min and diluted this with PPP to produce a specific platelet count (10, 50, 75 and 100 × 10^9/L) [25]. Peterson et al. centrifuged whole blood at 100 × g for 10 min, before diluting with PPP to achieve a standard platelet count of 50 × 10^9/L [40]. Bernhard et al. pelleted and washed platelets, before re-suspending them in PPP and adjusting “to similar counts” [38]. The International Society on Thrombosis and Haemostasis recommends centrifugation at 200 × g for 10 min with no brake to produce PRP [41]. These recommendations, to reduce red cell contamination and maintain platelet quiescence, derive from an adult study [42]. In CAT, PPP is often added to PRP to standardise platelet counts. The study by Haidl et al. suggests that in term neonates, PRP platelet counts do not influence thrombin generation parameters [25].

To date, no studies have evaluated the effect of premature platelets on neonatal haemostasis.

## The effect of extracellular vesicles on haemostasis

Extracellular vesicles (EVs) are nanoparticles (ranging from 50 to 1000 nm) released from cells [43], surrounded by a lipid bi-layer. A majority (> 70%) of plasma EVs are derived from platelets [44], typically released upon their activation [45]. EVs may play a role in haemostasis, increasing the phospholipid surface for the enzymatic reactions of the coagulation cascade and potentially increasing the local concentration of TF present [46]. TF bearing EVs originate from many cells, including endothelial cells and monocytes [47, 48]. Several studies have demonstrated an increase in the number of platelet-derived EVs and procoagulant EV activity in neonates compared with adults [37, 49–53].

CAT was used to evaluate the procoagulant effect of EVs in term cord blood compared with adults [21]. CAT was performed using *Thrombinoscope BV* PPP reagent (tissue factor and phospholipid) and microparticle (MP) reagent (phospholipid only). The MP/PPP ratio was used to evaluate the relative effect of TF-EVs on thrombin generation. TF-EVs had a greater impact on thrombin generation in neonates than adults. This increased procoagulant EV activity supports the possible compensatory role of EVs in the neonatal haemostatic system.

## CAT as a predictor of clinical bleeding in neonates

Standard clotting tests do not accurately predict the risk of bleeding [8, 10, 11]. Numerous studies have evaluated CAT as a predictor of bleeding in adults [54, 55], but few have in neonates. Peterson et al. found that CAT did not predict post-operative bleeding after cardiopulmonary bypass (CPB) [40]. Tripodi et al. found no difference in ETP measurements at birth, between VLBW infants that developed an IVH and those that did not [9]. Similarly, Neary et al. demonstrated no difference in any thrombin generation parameters between infants who developed a severe (or any) IVH and those that did not [56]. The current clinical application of CAT in neonates is limited by a lack of evidence to support CAT as a predictor of bleeding.

## CAT to evaluate haemostatic therapies in neonates

Sick neonates frequently receive blood products and haemostatic drugs, but few randomised controlled trials have evaluated their use in this population [34, 57]. Neonatal doses are frequently extrapolated from adult regimens, and guidelines are often consensus agreements. PlaNeT 2 has raised awareness of the potential harms of blood products in neonates [34]. Haemostatic drugs must be evaluated in neonates, given the differences in neonatal factor levels [2, 3]. CAT has been used as a pre-clinical tool to evaluate the potential haemostatic effects of drugs in neonates.

Cvirn et al. evaluated the anti-coagulant effect of Melagatran, a direct thrombin inhibitor, in neonatal cord blood and adult PPP [20]. While a similar concentration of Melagatran was required to prolong the lag time and time to peak in both groups, both ETP and peak thrombin were suppressed by over 50% using a much lower drug concentration in cord blood plasma than that required to achieve the same effect in adult plasma. These distinct patterns of sensitivity to the same anticoagulant drug highlight the variability in endogenous haemostatic pathway activity which exists between neonatal and adult plasma, detectable by CAT.

CAT assessed the effect of ex vivo addition of NovoSeven® (recombinant factor VIIa) or three-factor prothrombin complex (3f-PCC) (containing FII, FIX, FX, and a small amount of FVII) to PPP of term infants post CPB [58]. While NovoSeven® reduced the lag time only, 3f-PCC also significantly increased peak thrombin and velocity index, above pre-CPB levels.

Franklin et al. studied the effect of two “four-factor prothrombin complex concentrates” (4f-PCCs), one which contained FVII and the other FVIIa, in PPP from term infants who had undergone CPB [19]. While both concentrations increased the peak thrombin and velocity index, only the preparation containing FVIIa reduced lag time to pre-CPB levels. The lower dose of both drugs tested was sufficient to enhance thrombin generation in neonates.

The effect of NovoSeven® in umbilical cord blood and adult PRP was investigated [25], due to the high incidence of reported thrombotic adverse events in the neonatal population [59]. NovoSeven® altered clot dynamics; it did not alter ETP in either group but shortened the lag time and reduced the peak height in both groups, most significantly in the neonatal group. The effect of rFVIIa did not appear to be platelet dependent in vitro. Moreover, the dose response to rFVIIa was comparable between the neonates and adult PRP.

CAT cannot replace trials to evaluate the clinical effects of these drugs, but it may provide some insights into the relative effects of haemostatic therapies in supporting normal coagulation, at least in vitro.

## CAT in specific populations

### Infants undergoing cardiopulmonary bypass

Infants who require neonatal surgical correction of cardiac malformations with CPB are at high risk of post-operative haemorrhage, due to a dilution of coagulation factors, exposure to heparin anti-coagulation and activation of blood cells as a result of interactions with extravascular tissue and artificial tubing [60]. Neonates typically receive blood products and pro-coagulant drugs to overcome these challenges.

Two studies evaluated thrombin generation in neonates pre- and post-CPB (following the reversal of heparin and the administration of blood products) [19, 58]. Both demonstrated a prolonged lag time post-CPB and an increase in peak thrombin compared with pre-CPB samples.

Peterson et al. evaluated CAT in PRP compared with other coagulation assays, assessing heparin reversal and rebound effect, in neonates undergoing CPB [40]. CAT results were compared with thrombin-initiated fibrin clot kinetics (TFCK). Peak thrombin inversely correlated with high TFCK ratios (blood samples with the highest heparin activity).

### Factor VIII deficiency

Neonates with factor VIII deficiency can develop severe haemorrhage [61]. CAT evaluated the effect of factor VIII levels in neonates in cord blood PPP, with varying levels of factor VIII, anti-thrombin and TFPI [24].

Factor VIII depleted neonatal plasma showed a slight prolongation in lag time and time to peak, with no change in peak thrombin. In a neonate with confirmed factor VIII deficiency, the lag time and time to peak were slightly prolonged; however, the peak thrombin was reduced by 25%.

An increase in TFPI levels resulted in a prolonged lag time and time to peak, but had little effect on peak height or ETP. In contrast, an increase in the anti-thrombin level resulted in a large reduction in ETP (64%) and peak height (33%) but no change to the lag time. This study illustrated the mechanisms by which the inhibitory pathways impact on thrombin generation parameters in neonates.

### Cholestatic liver disease

Preterm infants are at risk of cholestasis due to prolonged parenteral nutrition use. Cholestasis reduces vitamin K absorption, and thus, vitamin K–dependent coagulation factors. It was hypothesised that infants with intestinal failure associated liver disease may have impaired thrombin generation [62]. CAT was performed in PPP at birth and day 30 in the presence of thrombomodulin. In spite of prolonged standard clotting tests in the liver disease group, there was no difference in ETP between groups at either timepoint.

## Strengths and limitations of CAT compared with alternative global coagulation assays

CAT is a useful tool to evaluate in detail the mechanisms of haemostasis in neonates, and to explore how these differ from adults. CAT evaluates for both hypo- and hypercoagulability [15] and has good intra- and inter-individual reliability [15]. Plasma CAT is a useful method of evaluating the function of specific plasma coagulation proteins.

CAT is only used as a research tool in neonates, not as a clinical assessment tool, and neonatal studies have derived from a small number of centres internationally. CAT requires the manual preparation of plasma and addition of reagents as described in Fig. 1. The reagents may be prepared on site or purchased from a manufacturer. Purchased reagents are costly, and CAT also requires specific instruments and software which would not be routinely available in clinical laboratories. There are challenges to the interchangeability of results between sites, particularly in relation to the pre-analytical steps and standardisation of reagents used, although studies have shown interchangeability between sites to be within acceptable ranges when standardised protocols are followed [63–65]. Currently, neonatal reference ranges or treatment algorithms that allow evidence-based prescribing of blood products do not exist for CAT. Moreover, CAT does not evaluate primary haemostasis or the effect of red or white cells, vascular endothelium or blood flow on secondary haemostasis [15].

Alternative global coagulation assays, such as thromboelastography (TEG) and rotational thromboelastometry (ROTEM), use viscoelastic techniques to evaluate clot formation [66]. These may be useful in overcoming some limitations of CAT (Table 2). TEG/ROTEM evaluate the fibrinolytic system and are performed in whole blood, thus assessing the effect of all cellular blood components, requiring less time and skill to prepare, and reducing the risk of iatrogenic platelet activation. Standard CAT in duplicate requires up to 320 µL of plasma, while TEG requires only 340 µL of whole blood per analysis [67]. The blood volume required limits the use of CAT in neonates. Several CAT studies are performed in umbilical cord blood, given the larger volumes available. However, there is some evidence of a procoagulant imbalance in cord blood compared with neonatal blood, which may limit its applications [68]. The intra-assay reliability is acceptable in TEG, even in VLBW infants [67], and neonatal TEG reference ranges exist for both term and preterm infants [69, 70].

**Table 2:** A comparison of CAT with viscoelastic assays (TEG/ROTEM) in neonates [13, 15, 71]

	Thrombin generation assays (CAT)	Viscoelastic assays (TEG/ROTEM)
Principle of assay	Assesses ability to generate thrombin (the key effector protease of the coagulation cascade) in platelet-rich or platelet-poor plasma Generation of fibrin clot occurs rapidly (and prior to exhaustion of the thrombin-generating capacity of plasma), and therefore, parameters of thrombin generation as opposed to clot formation may be more informative and more reflective of the overall complexity of the haemostatic balance The assay may be modified to examine specific components of haemostasis in isolation (e.g. APC sensitivity).While some modifications of this assay have been described, in general, CAT has not been widely used for assessing whole blood coagulation or fibrinolysis	Assesses fibrin clot formation and fibrinolysis in whole blood This will take into account the influence of plasma, platelets, leucocytes and red cells. As fibrinolysis is also assessed in parallel, this assay may have more immediate clinical applications in certain scenarios, such as in the management of major haemorrhage Individual components of blood coagulation cannot be assessed
Volumes required	The required volume will be dependent on the clinical/research scenario Only the plasma fraction of whole blood is utilised; thus, larger volumes of whole blood are typically required to yield the required plasma sample volume	Generally, a smaller volume is required as the entire whole blood sample is used
Timing of analysis, sample types, availability of results	Fresh or thawed batches of frozen plasma may be used Analysis can take up to 1 h to complete	Immediate analysis of whole blood samples only—not suitable for stored samples Assay completed and results available within minutes
Location of testing	Laboratory-based	Bedside point of care assay
Availability of neonatal reference ranges	No	Yes

## Conclusions

CAT studies have expanded our knowledge of crucial differences between neonatal and adult haemostasis and potential compensatory mechanisms in neonates. These present a narrative, that neonates, including preterm neonates, may have equivalent haemostatic potential or even be hypercoagulable compared with adults. This questions the practice of administering blood products and pro-coagulant drugs to non-bleeding neonates with deranged standard clotting tests.

Moving forward, CAT represents a useful research tool to evaluate haemostatic therapies and to expand our understanding of the intricacies of neonatal haemostasis. However, the role of CAT as a bedside clinical tool is limited, and this function may be better suited to whole blood assays such as TEG/ROTEM.

## Abbreviations

3f-PCC: Three-factor prothrombin complex concentrate
4f-PCC: Four-factor prothrombin complex concentrate
APTT: Activated partial thromboplastin time
CAT: Calibrated automated thrombogram
CPB: Cardiopulmonary bypass
ETP: Endogenous thrombin potential
EV: Extracellular vesicle
IVH: Intraventricular haemorrhage
MP: Microparticle
PPP: Platelet poor plasma
PRP: Platelet-rich plasma
PT: Prothrombin time
ROTEM: Rotational thromboelastometry
SGA: Small for gestational age
TEG: Thromboelastography
TF: Tissue factor
TFCK: Thrombin initiated fibrin clot kinetics
TFPI: Tissue factor pathway inhibitor
TM: Thrombomodulin
VLBW: Very low birth weight
